# Chitosan and cloxacillin combination improve antibiotic efficacy against different lifestyle of coagulase-negative *Staphylococcus* isolates from chronic bovine mastitis

**DOI:** 10.1038/s41598-018-23521-0

**Published:** 2018-03-23

**Authors:** María L. Breser, Verónica Felipe, Luciana P. Bohl, María S. Orellano, Paula Isaac, Agustín Conesa, Virginia E. Rivero, Silvia G. Correa, Ismael D. Bianco, Carina Porporatto

**Affiliations:** 1 Centro de Investigación y Transferencia (CIT-CONICET), Universidad Nacional de Villa María, Arturo Jauretche 1555, Ciudad Universitaria, Villa María, Argentina; 2 Instituto A.P. de Ciencias Básicas y Aplicadas, Universidad Nacional de Villa María, Arturo Jauretche 1555, Ciudad Universitaria, Villa María, Argentina; 3 Centro de Investigaciones en Bioquímica Clínica e Inmunología (CIBICI-CONICET), Facultad de Ciencias Químicas, Universidad Nacional de Córdoba, Córdoba, Argentina; 4 Centro de Excelencia en Productos y Procesos de Córdoba (CEPROCOR), Ministerio de Industria, Comercio, Minería y Desarrollo Científico Tecnológico, Córdoba, Argentina

## Abstract

Bovine mastitis affects the health of dairy cows and the profitability of herds worldwide. Coagulase-negative *staphylococci* (CNS) are the most frequently isolated pathogens in bovine intramammary infection. Based on the wide range of antimicrobial, mucoadhesive and immunostimulant properties demonstrated by chitosan, we have evaluated therapy efficiency of chitosan incorporation to cloxacillin antibiotic as well as its effect against different bacterial lifestyles of seven CNS isolates from chronic intramammary infections. The therapeutic effects of combinations were evaluated on planktonic cultures, bacterial biofilms and intracellular growth in mammary epithelial cells. We found that biofilms and intracellular growth forms offered a strong protection against antibiotic therapy. On the other hand, we found that chitosan addition to cloxacillin efficiently reduced the antibiotic concentration necessary for bacterial killing in different lifestyle. Remarkably, the combined treatment was not only able to inhibit bacterial biofilm establishment and increase preformed biofilm eradication, but it also reduced intracellular bacterial viability while it increased IL-6 secretion by infected epithelial cells. These findings provide a new approach to prophylactic drying therapy that could help to improve conventional antimicrobial treatment against different forms of bacterial growth in an efficient, safer and greener manner reducing multiresistant bacteria generation and spread.

## Introduction

Mastitis is the most prevalent and costly disease in dairy cattle around the world^1,2^. Although its incidence varies according to the country and the type of herd management, the economic losses caused by this pathology include costs above 10% of net income of dairy farms^1,3^. Coagulase-negative *staphylococci* (CNS) previously considered a minor or opportunistic pathogens, are currently the most predominant isolated bacteria in intramammary bovine mastitis in dairy cattle worldwide^4–7^. Remarkably, CNS have been associated with persistent infections and high resistance to antibiotic therapy^8–12^. These may be because CNS have higher mutation rate and permeability to the horizontal transference of virulence factors than other mastitis-associated pathogens^13–15^. Some authors suggest that CNS could act as a reservoir or transport of large variability of virulence factors into mastitis pathogens^13,16,17^. In view of the above facts, it is clear that a more effective therapy to CNS infections control seems necessary.

Bacterial growth in biofilms or at the intracellular levelly represents important virulence factors that may be associated with the persistence of intramammary infections^18–20^. Some authors have suggested that CNS biofilm may offer protection against different antimicrobial strategies to develop recurrent infections under physiological, metabolic and/or immunological stress environments^18–22^. Other suggest that bacterial intracellular maintenance on host cells is an important strategy that allows hiding as well as avoiding the recognition of host defense and antimicrobial therapies^20^.

Even when antibiotic therapy is the most efficient treatment to mastitis control, in many cases, infections cannot be fully resolved, generating bacterial resistance and presence of antibiotic residues in milk^23^. On the other hand, antibiotic prophylaxis during the drying period has had the highest cure rate of subclinical and chronic infections and has prevented the development of new intramammary infections during the dry and the peripartum period^24,25^. Nowadays, the most frequently used drying therapies for dairy cattle are cloxacillin, ampicillin and ceftiofur^26,27^. Prolonged antibiotic use increases selective pressure on bacterial populations and predisposes the microorganism to develop resistance^28,29^. However, cloxacillin resistance rates and efficacy against different bacterial growth mode isolated from mastitis have not been fully clarified. The high rate of bacterial resistance to traditional antimicrobials and the lack of development of new antibiotics by the pharmaceutical industry present a great problem not only in the veterinary field, but also in public health worldwide^28,29^. Based on the emergence of high antibiotic resistance in bovine isolates, it is necessary to incorporate innovative strategies to intramammary control and to prevent the development of multiresistant strains.

Chitosan is a natural cationic polymer with antimicrobial activity against a broad spectrum of pathogens^30,31^. Chitosan is biodegradable, biocompatible, bioadhesive, bioactive and non-toxic, and has been proposed to different therapeutic applications in a wide range of administration routes^30,31^. Antimicrobial activity of this biopolymer is based on its physical properties, and therefore it is more difficult to develop or transfer resistance^31^. Currently, the World Health Organization recommends the development of new therapies that minimize the use of antibiotics in food producing animals in order to prevent the generation and the spread of multiresistant bacteria.

Based on this, some countries have started to prohibit antibiotic uses as growth promoters and prophylactic treatment^32,33^. In the present study, we analyzed seven isolates from chronic bovine mastitis, which were identified as CNS and were refractory to different antibiotic therapies. All of them were able to grow in bacterial biofilms and intracellular form in a bovine mammary epithelial cell line. Our results show that chitosan addition to cloxacillin therapy significantly reduced antibiotic concentration to improve bacterial killing against planktonic cultures, preformed biofilms and intracellular growth, compared to conventional antibiotic treatment. These findings may contribute to understand the influence of different bacterial lifestyle and antibiotic resistance and provide bases for new strategies to be applied during the drying period to control persistent intramammary infections and prevent the development and spread of multiresistant bacteria.

## Results

### Antibiotic susceptibility of CNS isolates from chronic bovine mastitis

Isolates from chronic mastitis were tested for their susceptibility to different antibiotics frequently used in the veterinary clinic. In our study, penicillin, erythromycin, rifampicin, ampicillin, lincomycin and cefoxitin were tested^5^. Cefoxitin was used as an indicator of methicillin resistance^34^. Antibiogram tests showed that CNS isolates from chronic bovine mastitis (CM-CNS) exhibited 85%, 57% and 42% resistance to penicillin, ampicillin and methicillin, respectively (Table 1). On the other hand, 71%, 57% and 14% of chronic isolates showed resistance to erythromycin, lincomycin and rifampicin, respectively (Table 1). Notably, 85%, 71% and 57% of these isolations showed resistance to more than two, three and four antibiotics with different mechanisms of action (Table 1). Nevertheless, isolates from chronic mastitis presented different antibiotic resistance-patterns: six out of seven chronic isolates showed antibiotic multiresistance, although one of them that was susceptible to all of the antibiotics tested (Table 1). Based on susceptibility criteria by conventional antibiograms it was not possible to discriminate a clear pattern of multiresistance.

**Table 1:** Antibiotic susceptibility in CNS isolates from chronic bovine mastitis. R, resistant; S, susceptible. AMP, ampicillin; PEN, penicillin G; FOX, cefoxitin; ERY, erythromycin; RYF, rifampicin; LIN, lincomycin. Percentage of resistance to each antibiotic is shown in the last row.

Isolates	AMP	PEN	FOX	ERY	RIF	LIN
CM-CNS1	S	R	R	S	S	R
CM-CNS2	R	R	S	R	S	R
CM-CNS3	R	R	R	R	S	R
CM-CNS4	R	R	R	R	S	S
CM-CNS5	S	R	S	R	S	S
CM-CNS6	R	R	S	R	R	R
CM-CNS7	S	S	S	S	S	S
Resistant (%)	57	85	42	71	14	57

### Cloxacillin effect on planktonic cultures and preformed bacterial biofilms of CNS isolates from chronic bovine mastitis

As far as we know, the dry-period prophylaxis treatment shows the major cure-rate for chronic infections. On this basis, we analyzed cloxacillin susceptibility of our chronic isolates. Taking into account β-lactamase CLSI-breakpoints for CNS to Minimal Inhibitory Concentration (MIC)^34^, six of seven isolates were cloxacillin-resistant, while CM-CNS7 isolate was cloxacillin-susceptible (Table 2**)**. In our work, all isolates from chronic mastitis grew up in biofilm forms on abiotic surfaces (Supplementary Fig. 1). In order to understand if biofilm influence the cloxacillin resistance, we evaluated the Minimal Bactericidal Concentration (MBC) required against planktonic cultures (P-MBC) and preformed biofilms (B-MBC). We found that the cloxacillin concentration required to kill bacterial cells inside biofilms was higher than the amount needed for planktonic cultures. In fact, bacteria growing inside the biomass of biofilms needed between 16- to 128-fold higher cloxacillin concentration than the same bacteria growing in planktonic lifestyle, even with a susceptible isolate (Table 2). Strikingly, when grown inside biofilms, the CM-CNS7 isolate, which was sensitive to all antibiotics tested including cloxacillin, did not show any difference from others multiresistant CNS isolates (Table 2). On the contrary, in the biofilms form the CM-CNS7 isolate needs higher concentrations of antibiotic than other chronic isolates. Together, our data suggest that the growth of bacteria within biofilms mass could be determinant in the failure of antibiotic therapies, even in susceptible bacteria (defined by conventional methods). This suggests that this type of growth mode cannot be ignored in bacterial susceptibility tests.

**Table 2:** Cloxacillin susceptibility from planktonic cultures and preformed biofilms of CNS isolates from chronic bovine mastitis. Cloxacillin Minimal Inhibitory Concentration (MIC). Cloxacillin CLSI-breakpoint for CNS were considered ≥0.5 µg/mL. Cloxacillin Minimal Bactericidal Concentration on planktonic cultures (P-MBC), Cloxacillin Minimal Bactericidal Concentration on preformed biofilms (B-MBC). Bactericidal ratio between preformed biofilms and planktonic cultures (B-MBC/P-MBC).

	MIC [µg/mL]	P-MBC [µg/mL]	B-MBC [µg/mL]	B-MBC/ P-MBC
CM-CNS1	1	4	256	64
CM-CNS2	2	8	128	16
CM-CNS3	1	4	256	64
CM-CNS4	2	4	128	32
CM-CNS5	1	8	256	32
CM-CNS6	2	4	256	64
CM-CNS7	0.250	4	512	128

### Chitosan antimicrobial activity on planktonic cultures and preformed biofilms of CNS isolates from chronic bovine mastitis

**Figure 1:**
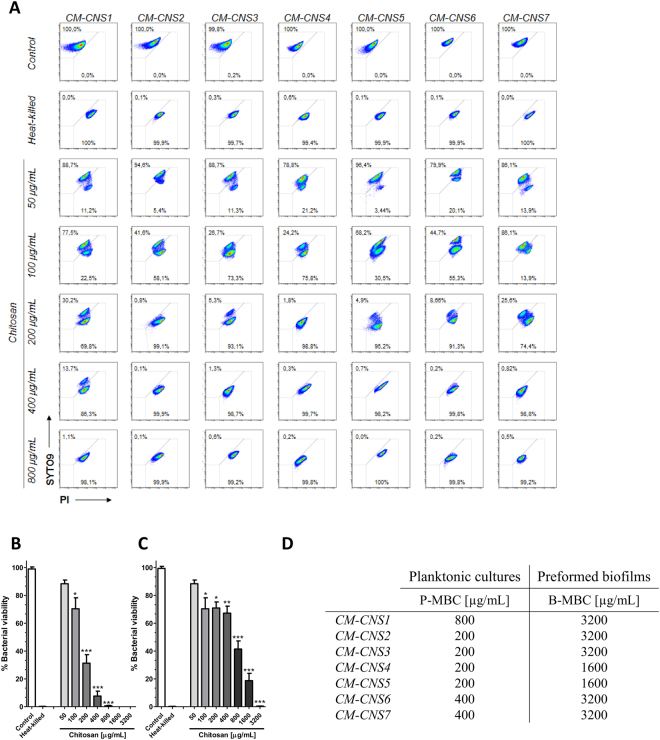
Antimicrobial activity of chitosan against CNS isolated from chronic intramammary infections. CNS growth in planktonic and biofilms forms were treated with different concentration of chitosan. (**A**) Bacterial viability of planktonic cultures were analyzed by flow cytometry using SYTO9 and PI dyes. (**B**) Bar graph shows chitosan effect on planktonic growth of CM-CNS isolates by flow cytometry. (**C**) Bar graph shows chitosan effect on preformed biofilms of CM-CNS isolates by flow cytometry. (**D**) Data table represents MBC in planktonic and biofilms growth by plate count assay. These experiments were performed three independent times with three biological replicates and the data are represented by nine independent replicates. Data are shown as mean ± SEM. The *p* values were obtained using two-way ANOVA followed by Bonferroni post-hoc analysis. P values *< 0.05, **< 0.01 and ***< 0.001 were considered significant.

Cloxacillin has shown to reduce effects over bacterial biofilms from chronic infected animals. On the contrary, chitosan is a biopolymer with a wide range of antimicrobial properties^30,31^. Antimicrobial activity of chitosan was evaluated by flow cytometry and plate counts. Bacterial membrane integrity was analyzed using the SYTO9 dye that can be incorporated into all cells (both alive and dead) and propidium iodide (PI) dye that can only be incorporated into dead cells (Fig. 1A–C). We found that chitosan presented a strong antimicrobial activity not only on planktonic cultures (Fig. 1A,B,D), but also on preformed biofilms (Fig. 1C,D), in a dose-dependent manner. Chitosan concentration required for bacterial killing inside preformed biofilms was from 4 to 16 times higher than for the planktonic cultures of the same isolate (Fig. 1D). In our study, chitosan presented important bactericidal activity on CNS isolates from chronic bovine mastitis that was independent of the antibiotic resistance pattern.

### Effect of cloxacillin and chitosan combination on planktonic cultures and bacterial biofilms of CNS isolates from chronic bovine mastitis

Next, we evaluated antimicrobial properties of the combined- chitosan and cloxacillin on planktonic cultures and preformed biofilms of CNS isolates. We found that chitosan addition to cloxacillin drastically reduced the antibiotic concentration needed for bacteria killing in different lifestyles. The combined treatment involved an antibiotic 16- to 64- fold reduction in planktonic cultures (Table 3), and 5- to 16- fold in preformed biofilms (Table 3), compared with the antibiotic alone. These results demonstrate that chitosan addition to cloxacillin formulation increased the efficacy of the antibiotic effect reducing the required concentration.

**Table 3:** Chitosan addition to cloxacillin treatment reduces antibiotic concentration needed to eliminate CNS in planktonic cultures and preformed biofilms.

	Planktonic cultures			Preformed biofilm		
	P-MBC [µg/mL]			B-MBC [µg/mL]		
	Clox.	Clox. + Ch.	Clox/Clox. + Ch.	Clox.	Clox. + Ch.	Clox/Clox. + Ch.
CM-CNS1	4	0.125	32	256	32	8
CM-CNS2	8	0.125	64	128	24	5
CM-CNS3	4	0.125	32	256	48	5
CM-CNS4	4	0.250	16	128	24	5
CM-CNS5	8	0.250	32	256	48	5
CM-CNS6	4	0.125	32	256	32	8
CM-CNS7	4	0.250	16	512	32	16

CNS were cultured in the presence of medium or different concentrations of cloxacillin [512–0.062 µg/mL] chitosan [200 µg/mL] or cloxacillin and chitosan combined for 24 h at 37 °C. (A) Planktonic CNS cultures were exposed to different conditions and P-MBC were determined in each case. (B) CNS preformed biofilms were exposed to different conditions and B-MBC were determinate in each case. Relation between MBC in cloxacillin presence and MBC in cloxacillin and chitosan combined presence was determined. These experiments were performed three independent times with three biological replicates.

### Chitosan and cloxacillin combination inhibited new bacterial biofilms formation and eradicated preformed biofilms

**Figure 2:**
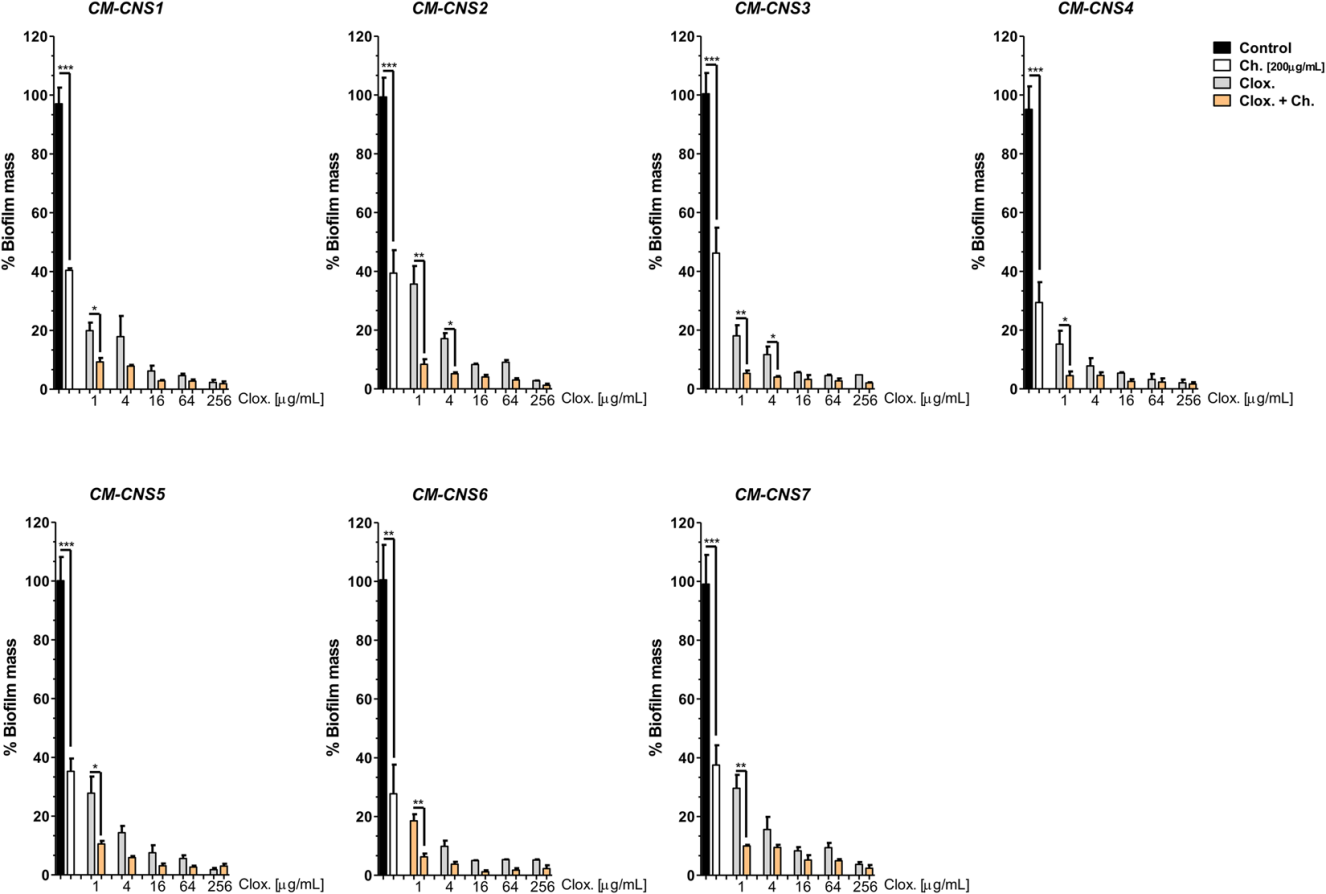
Chitosan prevents CNS bacterial biofilms formation itself and its addition to cloxacillin can help to avoid them. Bacterial cultures were seeded in 96-well plates in the presence of medium of different cloxacillin [1, 4, 16, 64, 256 µg/mL], chitosan [200 µg/mL] or cloxacillin concentration and chitosan combinations for 24 h at 37 °C. Biofilm biomass were evaluated by CV and biofilm generated in control condition (medium) was considered as 100% of biomass. These experiments were performed three independent times with three biological replicates and the data are represented by nine independent replicates. Data are shown as mean ± SEM. The p values were obtained using two-way ANOVA followed by Bonferroni post-hoc analysis. P values *< 0.05, and **< 0.01 and ***< 0.001 were considered significant.

Biofilm formation represents an important mechanism of virulence in antibiotic resistance and tolerance. Agents that may help to prevent biomass formation or eradicate preformed biomass could be very useful as a complement of antibiotic therapy. To evaluate this, we seeded a bacterial inoculum on a flat polystyrene plate in the presence of different concentrations of cloxacillin alone or combined with 200 µg/mL chitosan for 24 h (Fig. 2). As can be seen, chitosan and cloxacillin *per se* significantly inhibited bacterial biofilm formation. However, the combined treatment completely inhibited biofilm formation at low cloxacillin concentrations (Fig. 2).

**Figure 3:**
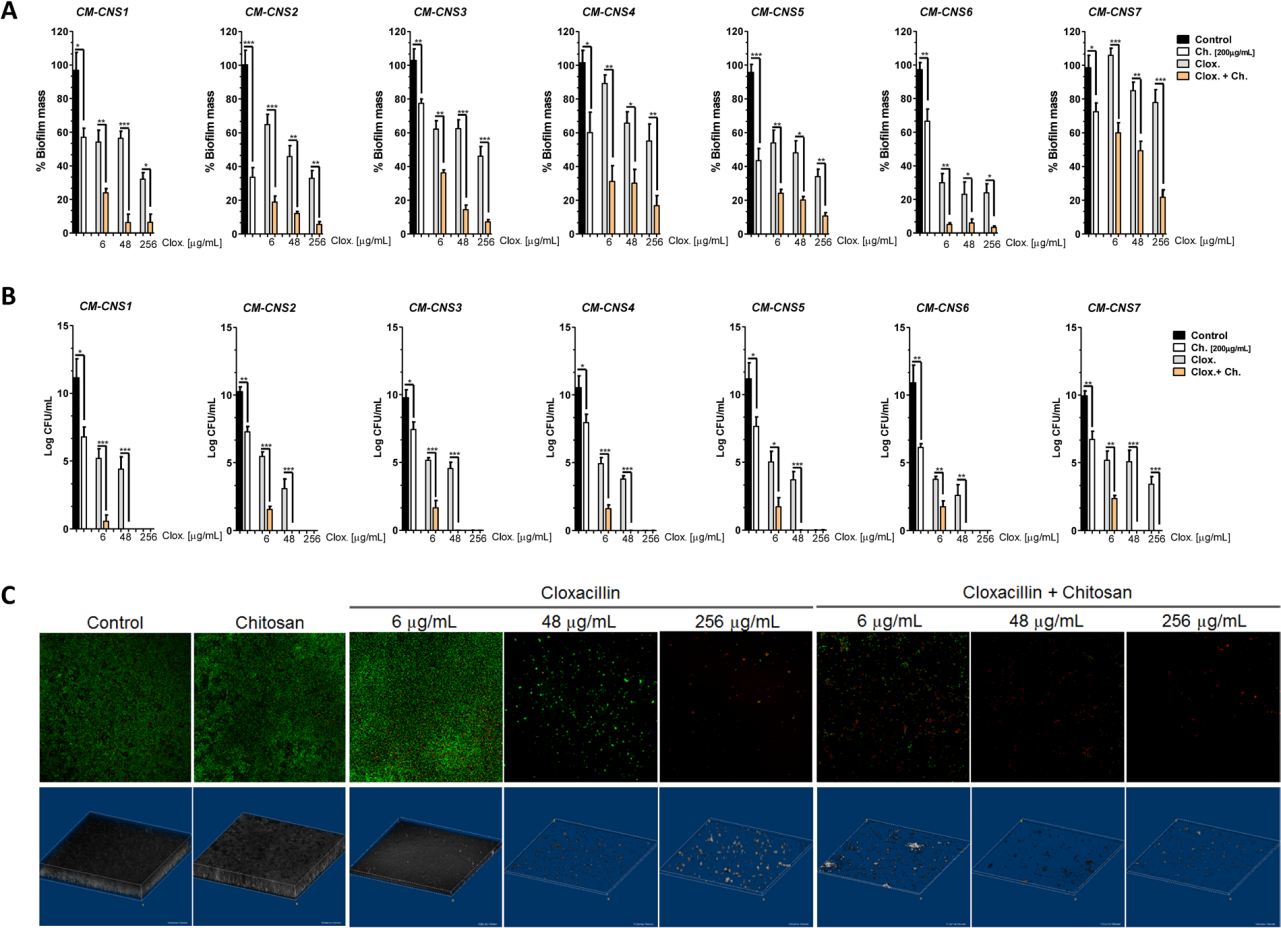
Chitosan and cloxacillin combination can eradicate preformed biofilms and increase bacterial killing inside them. CNS isolates from chronic intramammary infections were grown in 96-well plate for 24 h at 37 °C. Preformed biofilms were washed with PBS and incubated in the presence of medium or different concentration of cloxacillin [6, 48, 256 µg/mL], chitosan [200 µg/mL] or cloxacillin and chitosan combined for 24 h at 37 °C. Biofilm biomass and viable cells were evaluated by CV, plate count and CLSM. (**A**) Cristal Violet evaluation and biofilm generated in control condition (medium) was considered as 100% of biomass. (**B**) Bacterial Viability was evaluated in plate count (CFU/mL). (**C**) Bacterial viability and biofilm 3D structure were evaluated by staining with SYTO9 and PI dyes and analyzed by CLSM microscopy. CLSM images belong to CM-CNS7 isolation which was taken as representative. The other isolations showed similar efficiency results. These experiments were performed three independent times with two biological replicates and the data are represented by six independent replicates. Data are shown as mean ± SEM. P values were obtained using two-way ANOVA followed by Bonferroni post-hoc analysis. P values *< 0.05, **< 0.01 and ***< 0.001 were considered significant.

In order to determine the effect of single or combined treatments on mature preformed bacterial biofilms, CM-CNS were incubated under different conditions. Biofilm biomass, three-dimensional structure and cell viability were analyzed using crystal violet (CV) assay, plate count and confocal scanning laser microscopy (CSLM). Our results show that while the addition of cloxacillin and chitosan alone to preformed biofilms significantly reduced biofilm biomass (Fig. 3A,C) and bacterial viability count (Fig. 3B), the combined treatment dramatically improved the activity of individual components (Fig. 3A–C). Although cloxacillin reduced preformed biofilm biomass and bacterial viability, chitosan addition increased the efficacy of the antibiotics, reducing the cloxacillin concentration required (Fig. 3). Interestingly, the three-dimensional biofilm structure and the bacterial viability by CLSM demonstrated that only chitosan addition was able to reduce the thickness and compaction of the biomass structure and induced a slight reduction of bacterial viability, compared with the control condition (Fig. 3C and Supplementary Fig. 2). Together, the combined treatment could not only help to prevent the development of new foci of bacterial colonization, but also to eradicate preformed biofilms and induce bacterial killing.

### Chitosan and cloxacillin combination may improve bacterial clearance of CNS infected mammary epithelial cells

**Figure 4:**
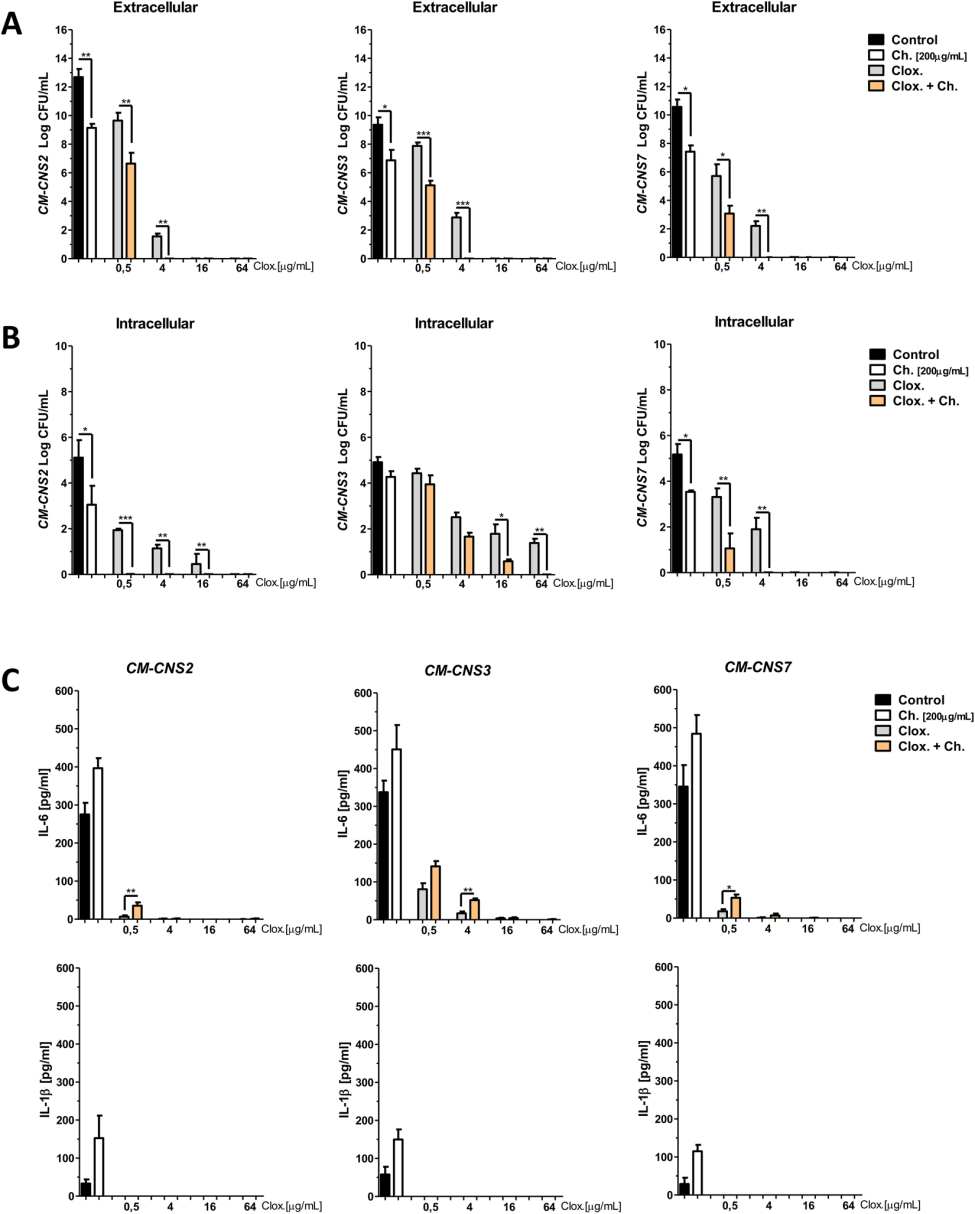
Chitosan and cloxacillin combination can reduce extracellular and intracellular MAC-T infection. MAC-T cells were infected for 2 h with CNS in a MOI 10. After that, MAC-T cells were cultured in the presence of medium or different concentrations of cloxacillin [0.5, 4, 16, 64 µg/mL], chitosan [200 µg/mL] or cloxacillin and chitosan combined for 24 h. (**A**) Graphs bar represent bacteria viability in extracellular supernatants (CFU/mL). (**B**) Graphs bar represent bacteria viability in intracellular MAC-T (CFU/mL). (**C**) Cytokines measured in culture supernatants of MAC-T cells treated with different conditions analyzed by ELISA. These experiments were performed two independent times with three biological replicates and the data are represented by six independent replicates. Data are shown as mean ± SEM. The p values were obtained using one-way ANOVA followed by Bonferroni post-hoc analysis. P values *< 0.05, **< 0.01 and ***< 0.001 were considered significant.

In this work, we also evaluated the antimicrobial effect of chitosan, cloxacillin and their combinations on the infection of bovine mammary epithelial cells (MAC-T). CM-CNS isolates used for MAC-T infection were selected based on biofilm biomass production. The viability of extracellular and intracellular bacteria was assessed after 24 h of treatment by plate count (Fig. 4A,B). Although chitosan and cloxacillin addition reduced bacterial viability in culture supernatants, the combined treatment significantly decreased the antibiotic concentration needed to perform extracellular and intracellular bacterial clearance (Fig. 4A,B). Cloxacillin addition to infected MAC-T cell cultures reduced the bacterial burden in the extracellular environment (in a dose-dependent manner), but the antibiotic effectively controlled intracellular infection on MAC-T cells only at the high concentrations (Fig. 4A,B). Differences in cloxacillin concentrations required to kill intra and extracellular bacteria suggest that intracellular survival might represent an important virulence mechanism associated to chronic infections. Interestingly, although single cloxacillin and chitosan *per se* addition may reduce bacterial viability, combined treatment can significantly diminish the antibiotic concentration needed to perform extracellular and intracellular bacterial clearance (Fig. 4A,B). On the other hand, we observed that chitosan induces a slightly increase in IL-6 and IL-1β proinflammatory cytokines secretion in culture supernatants of infected MAC-T cells, while chitosan combined with cloxacillin induces a slightly increase in IL-6 (Fig. 4C). We found that the combined therapy could significantly improve the extracellular and intracellular bacterial clearance, compared to the single antibiotic treatment. Moreover, based on our findings we could suggest that chitosan could not only act as antimicrobial agent against different bacterial lifestyles, but it could also contribute to epithelial cell response against CNS.

**Figure 5:**
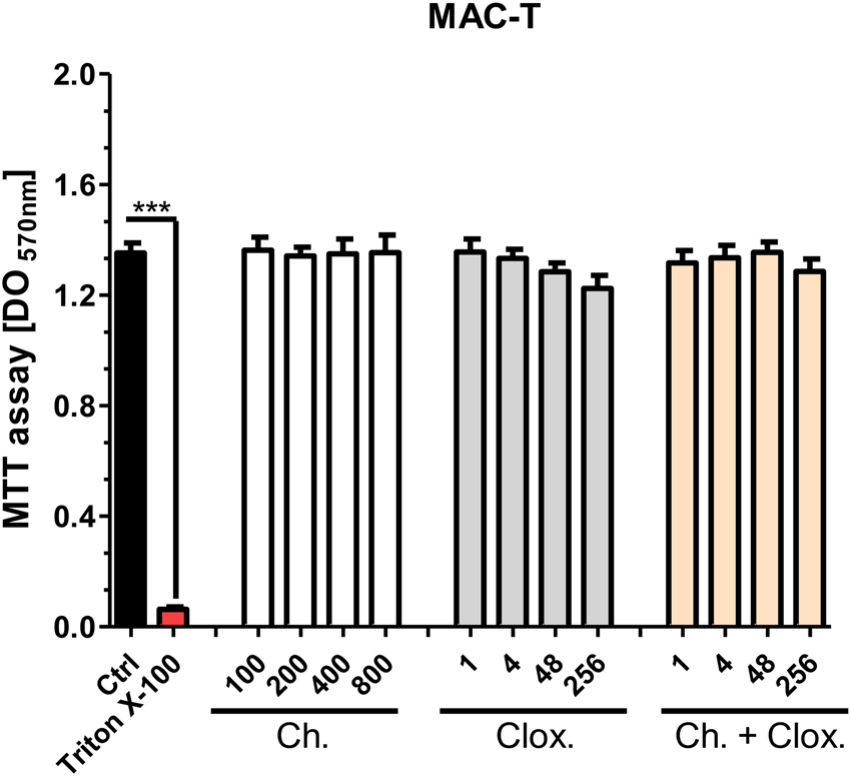
Cloxacillin, chitosan or their different combinations not affect MAC-T viability. MAC-T cells were cultured in the presence of medium or different concentrations of chitosan [100, 200, 400, 800 µg/mL], cloxacillin [1, 4, 48, 256 µg/mL] or cloxacillin [1, 4, 48, 256 µg/mL] and chitosan [200 µg/mL] combined for 48 h. Cell viability was assessed by the MTT assay. Graph bars represent MAC-T cells viability in the presence of different conditions. These experiments were performed three independent times with three biological replicates and the data are represented by nine independent replicates. Data are shown as mean ± SEM. The p values were obtained using one-way ANOVA followed by Bonferroni post-hoc analysis. P values ***< 0.001 were considered significant.

Finally, the viability of MAC-T cells was evaluated by MTT assay in cultures with medium, cloxacillin [1, 4, 48, 256 µg/mL], chitosan [100, 200, 400, 800 µg/mL] or different combinations for 48 h. We found that cell viability was not affected by any of the conditions tested of cloxacillin, chitosan or their different combinations (Fig. 5). Taken together, our data suggest that chitosan added to antibiotic drying therapy could be a safe alternative, which could not only improve bacterial clearance in different lifestyles, but also stimulate the defense of infected mammary epithelial cells.

## Discussion

The lack of development of new antibiotics and the exposure to sub-optimal concentrations could be associated with a reduced treatment efficacy and resistance development. Designs of new treatments should be generated through searching for new systems that take into account different forms of growth, avoiding falling into the post-antibiotic era, and the effects of not having useful antibiotics to treat resistant bacteria. Bacteria easily develop different mechanisms and virulence factors that allow adapting to the environment in which they live. Unfortunately, the speed at which these adaptations occur poses a great challenge for the pharmaceutical industry worldwide that needs to respond more efficiently and faster. In fact, studies in different countries have reported higher rates of resistance to antibiotics frequently used to mastitis control^8,9,35,36^. Empirical practice and uncontrolled use may be relevant risk factors for chronic or recurrent infections due to multiresistant and host-adapted selection strains^33^.

In recent years CNS have begun to represent pathogens with the highest antibiotic resistance not only in animals but also in human infections^37^. Reports on the growing importance of CNS as mastitis pathogens have increased in Argentina and throughout the world^38–40^. CNS have been described in persistent, incurable and chronic infections intimately associated with tissue damage, decreased milk quality and a higher rate of antibiotic multi-resistance compared to other pathogens associated with mastitis^5,7,19,20,41,42^. Noteworthily, the methicillin resistance observed in our study was similar to data observed in bovine mastitis isolates^43,44^, but higher than other reports^8,11,12,22^. Furthermore, we found that chronic isolates presented a significantly higher macrolide and lincosamide resistance than others reports^8,11,22,43^. The cumulative evidence obtained in recent years showed significant problems associated with the chronic use of antibiotics in production animals worldwide. In the last years, reports show high methicillin-resistance in CNS isolated from beef meat, poultry farm and chickens^45,46^. These findings highlight the potential risk posed by the consumption of food contaminated with microorganisms with this characteristic and the high impact they can have on public health.

Virulence factors associated with the growth of bacteria may be important in the clinical failure of different treatments^20,47,48^. Indeed, recurrent failure of antibiotic therapies has been associated with bacterial isolation without antibiotic-resistance genes, suggesting that virulence factors such as different bacterial growths, might play a key role in their survival^20,22,47,49^. In fact, bacterial growth in biofilms or intracellular forms may induce a complex mechanism of recalcitrance towards antibiotics, which is associated with different mechanisms of antibiotic resistance^20,47^.

More than 85% of CNS isolated from bovine mastitis can grow in the biofilm form^18^, and in Argentinean dairy cattle this ability is present in more than 97% of isolates^22,40^. All the CNS isolated from chronic bovine mastitis studied here presented strong ability to grow in biofilm form. In our work, we found that planktonic cultures needed a significantly lower cloxacillin concentration than the same bacteria that were growing in biofilm form. In agreement with our data, other authors have shown that CNS planktonic cells from bovine mastitis with strong and weak biofilm phenotype have the same pattern of antibiotic susceptibility, but when grown in biofilm form, a significantly higher resistance was detected^19,42,50^. Interestingly, a strong association between the persistence of CNS intramammary infections, *in vitro* biofilms strength and antimicrobial resistance has been proposed^18,19,42,49^. It seems that properties of bacterial biofilm lifestyle is clearly different from free-living bacterial cells, but interestingly, bacteria from biofilms exhibit emergent properties that are not predictable from planktonic bacteria studies^47,50^. However, the biofilm mode of growth, its relationship to antibiotic resistance, and how it affects such resistance have not been fully elucidated^51^.

Some studies have compared planktonic MIC to minimum biofilm eradication concentrations (MBEC) or biofilm minimal inhibitory concentration (BMIC)^19,50^. Still, the impact of antibiotic treatment over bacterial biofilm viability deserves further research^52^. In our work, we compared cloxacillin bactericidal concentration on CNS from chronic bovine mastitis isolates in planktonic, biofilm and intracellular lifestyle. The presence of biofilms led to a 16- to 128-fold cloxacillin MBC increase, compared to planktonic cells, while intracellular infection produced a 4- to 32-fold cloxacillin MBC increase, compared to planktonic cells. Remarkably, antibiotics for the routine treatment of staphylococcal infections are generally evaluated to eliminate planktonic cells, while biofilm and intracellular bacterial growth are not being considered^20,47^.

Current efforts are directed toward finding broad spectrum antimicrobial compounds mainly associated to the treatment of bacteria, which could be useful to control and prevent the development and spread of infections. Chitosan shows antibacterial, antifungal, mucoadhesive and biodegradability properties, while it has been reported that this polymer increments intracellular trafficking and tissue regeneration without toxic effects on living tissues^30,31^. Different chitosan formulations have shown important antibiofilms properties against a wide range of pathogens^53–55^. Our data shows that chitosan and cloxacillin combination was more effective on different growth mode than their single counterparts were, reducing cloxacillin concentration required to bacteria killing. Notably, these combinations demonstrated a highly effective action capable of inhibiting biofilm formation and eradicating preformed bacterial biofilms, compared to their single counterparts. In agreement with these findings, some reports carried out on different bacterial genera, demonstrated that chitosan combined with antibiotics presents an important antibacterial activity to planktonic and biofilm bacterial lifestyle^56–58^. In fact, it has been reported that very low molecular weight chitosan and tilmicosin combined not only had synergic effect against planktonic and preformed biofilms of *S. aureus*, but also could reduce bacterial burden in intramammary infection in a murine model^58^. Accordingly, in a model of urinary tract infection the combination of chitosan and ciprofloxacin was able to eradicate more efficiently the infection and prevent recurrent episodes of bacteriuria caused by uropathogenic *Escherichia coli*^59^. This study also showed that animals treated with chitosan formulations, presented greater restoration of the genital tract epithelium integrity^59^. Recently, it has been reported that intramammary applications of chitosan hydrogels during the drying period, produced an improvement in the involution of the mammary gland and a stimulation of the innate immunity after calving^60^.

In summary, our results demonstrate that chitosan and cloxacillin combination reduced the antibiotic concentration required for bacterial killing in planktonic cultures, preformed biofilms and infected bovine mammary epithelial cells. Furthermore, chitosan incorporation not only improved intramammary bacterial clearance, but also led to a slight pro-inflammatory cytokine secretion after infection. To provide optimal protection against pathogens, the mammary gland immune system needs to be activated. Specifically, IL-6 is the third master proinflammatory cytokine and one of the key mediators of the “acute-phase response” in inflammation^61^. Production of these *in vivo* cytokines could be very useful to increase the recruitment and activation of immune cells. The chronologically coordinated induction of their synthesis at the site of inflammation is crucial for an effective inflammatory response, including pathogen clearance, wound healing, and return to the normal state^61^.

These data suggest that the addition of chitosan to antibiotic therapy could significantly improve the prognosis of chronic and persistent intramammary infections. Further studies in clinical trials on chronic infections carried out during the drying period should be conducted to validate *in vitro* results. Our findings could be a promising green and safe alternative for the complete cure of remaining infections during the drying period, as well as for the prevention of new infection development during the peripartum period.

## Materials and Methods

### Bacterial isolates, growth conditions and reagents

We use a collection of 209 *Staphylococcus* spp. isolates obtained from 10 independent dairy farms located within a 65 km radius of Villa María city (a major dairy producing area of Argentina). Animals with intramammary infections were followed for a 6 to 8-month period. Each chronic isolation was obtained after three independent identifications, carried out every 15 days, which represented a clinical intramammary infection for more than 45 days. Only seven isolates, obtained from four dairy farms, could be classified as chronic and were used in this study. These experiments were performed under the supervision and approval of the Institutional Ethics Committee for experiments with animals of the Universidad Nacional de Villa María. All assays were conducted in accordance with international guidelines and regulations for the use and handling of pathogenic microorganisms.

Species identification were previously confirmed by biochemical tests, MALDI-TOFF assay and 16 S sequencing test^40^. All chronic isolates were identified as CNS. The isolates were called chronic mastitis (CM)- CNS CM-CNS1, CM-CNS2, CM-CNS3, CM-CNS4, CM-CNS5, CM-CNS6 and CM-CNS7. *Staphylococcus aureus* (*S. aureus*) ATCC 29213, ATCC 25923 and Methicillin-Resistant *S. aureus* (MRSA) ATCC 43300, S. *epidermidis* ATCC 12228 and ATCC 35984 were used as standard control of assays in each case. The bacterial isolates and reference strains were stored at −80 °C, in a nutrient broth containing 20% of glycerol. Inoculum was prepared in Trypcase Soy Broth (TSB) at 37 °C 18 to 24 h prior to assay. The inoculums were adjusted using DensiCHEK Plus (BioMérieux Inc, Marcy l’Etoile, France) according to McFarland scale values and corroborated by plate counting.

Low molecular weight chitosan 50–90 kDa powder with ≥85% of deacetylation, cloxacillin antibiotic powder, Triton X-100, Crystal violet (CV) and 3-(4,5-Dimethylthiazol-2-yl)-2,5-Diphenyltetrazolium Bromide (MTT) were purchased to Sigma-Aldrich (Sigma-Aldrich, St. Louis, MO, USA). All the antibiotics disks, nutrient agar and nutrient broth were purchased from Britania (CABA, BA, Argentina).

### Susceptibility Testing

Antimicrobial susceptibility was determined by the standard disk diffusion method according to the Clinical and Laboratory Standards Institute (CLSI) guidelines^34^. All the bacterial strains were recovered on a fresh nutrient TSA 18–24 h prior to antimicrobial test. The direct colony suspension method was used to prepare bacterial suspension of 0.5 McFarland turbidity (CLSI). Bacterial suspensions were swabbed uniformly across a Müller-Hinton agar plate and antibiotic disks were placed on the surface of the agar. The antibiotic disks were placed on the agar surface and the lecture of diameters of inhibitions were collected 18 h after. Results were interpreted as either sensitive or resistant according to the inhibitory zone diameters around the disks using CLSI breakpoints^34^. Antibiotic disks assayed to antimicrobial tests included: penicillin (PEN), ampicillin (AMP), cefoxitin (FOX), erythromycin (ERY), rifampicin (RIF) and lincomycin (LIN). Cefoxitin disk was used as an indicator of methicillin susceptibility. All antimicrobial agents were chosen with regard to their relevance in mastitis therapy. *S. aureus* ATCC 25923 and *S. aureus* MRSA were used as quality control of microbiological assays.

### MIC and MBC assay

Minimum Inhibitory Concentration (MIC) and Minimum Bactericidal Concentration (P-MBC) of components against planktonic bacteria was preformed using microdilution assay according to CLSI guidelines^34^. Briefly, bacterial suspensions containing 5 × 10^5^ CFU/mL were cultured for 24 h at 37 °C into 96-wells bottom-plate (Deltalab, Barcelona, Spain) in presence of the different concentrations of cloxacillin, chitosan or combinations of them. MIC and MBC were performed in the Muller-Hinton Broth (MHB) at an initial concentration of 512 µg/mL of cloxacillin and 3200 µg/mL of chitosan, and then serially diluted. To determine P-MBC, each wells with equal and greater MIC in each case was seeded in TS-Agar (TSA) for plate count. Viable bacteria were evaluated 24 h after seeded.

### Flow cytometry viability assay

Bacterial suspensions were grown in the presence of TSB medium (Control) or in different concentrations of chitosan [800, 400, 200, 100, 50 µg/mL] for 24 h at 37 °C. Viability evaluation was performed using the LIVE/DEAD BacLight Bacterial Viability Kit staining (Invitrogen, ThermoFisher Scientific, CA, USA), according to the manufacturer’s instructions. Bacterial viability is carried out by SYTO9 and propidium iodide (PI) dyes which determine cell membrane integrity. SYTO9 dye can be incorporated to live and dead bacterial cells and can be useful to determine the total cells population, while PI dye is commonly used for identifying dead cells which present disrupted membranes. When both dyes are present, PI increase the intensity collected in FL3 filter and generates a bleaching effect of SYTO9 fluorescence intensity. Bacterial suspensions were acquired on an ACCURI C6 (BD Biosciences, San Diego, CA, USA) flow cytometer and the data were analyzed using FlowJo software (Tree Star, OR, USA).

### Biofilm inhibition assay

One hundred microlitres of 1 × 10^7^ CFU/mL of bacterial suspension were cultured in the presence of TSB medium (Control), chitosan [200 µg/mL], cloxacillin [512, 256, 128, 64, 32, 16, 8, 1 µg/mL] or combinations of chitosan with different concentrations of cloxacillin in 200 uL of final volume for 24 h at 37 °C. After that, bacterial supernatants were discarded and each individual wells were washed twice with sterile PBS and staining with a CV [0.5% w/v]. Excess colorant was washed and dried for 24 h. Colorant bound to each well was resuspended with 200 µL of 96% alcohol per well. After 20 min of incubation at room temperature, 100 µL of each well was transferred to a new 96-well plate and the absorbance of the eluate was measured at 590 nm using microplate spectrophotometer reader Multiskan GO (ThermoFisher Scientific).

### Bactericidal activity and biomass evaluation on preformed biofilms

One hundred microlitres of 1 × 10^7^ CFU/mL of bacterial suspension in TSB were added to individual wells of flat polystyrene microtitre plates. The final volume of 200 µL was completed with TSB medium. Plates were statically incubated for 24 h at 37 °C to allow cell bonding and biofilm formation. Supernatants were then discarded and the biofilms were washed twice with sterile PBS to remove non-adherent bacteria. The preformed biofilms were treated with different concentrations of cloxacillin, chitosan and its respective mixture for 24 h at 37 °C. After that, the biofilm mass was evaluated by CV staining. Bacterial viability cultured within biomass of biofilms (B-MBC) were evaluated by plate count, after desegregating with 0.1% of Triton X-100 before serial dilution^56^.

### Confocal scanning laser microscopy (CSLM)

Biofilms were grown overnight in TSB in flat 8-chamber plate Nunc Lab-Tek^TM^ Chamber Slides (ThermoFisher Scientific) for 24 h at 37 °C. After that, the medium was removed, and the biofilms were washed twice with sterile PBS to remove non-adherent cells. Bacterial inside biofilms were stained with LIVE/DEAD BacLight Bacterial Viability Kit staining, according to the manufacturer´s instructions. Briefly, biofilms were incubated 15 min in dark, after that these were washed twice with sterile PBS to remove the excess colorants. The controls biofilms were approximately 30 to 50 µm thick, and Z-slices were obtained every 0.5 microns. All confocal images and stacks of images were obtained at the Microscopy Center of Universidad Nacional de Córdoba. Each individual chamber was analyzed in a confocal laser scanning microscope (CLSM) Leica DM6000 CS (Leica Microsystems, Wetzlar, Germany), using a 60x oil immersion upright objective. The images and stacks of images were analyzed using the FIJI-IMAGEJ analysis software.

### Intracellular and planktonic bacteria in MAC-T cells co-cultures

MAC-T cell line from bovine mammary alveolar cells were maintained in a DMEM GlutaMAX™ medium (Thermo Fisher Scientific) supplemented with 1 μg/mL hydrocortisone (Fada Pharma, CABA, BA, Argentina), 5 μg/mL bovine insulin (Betasint, CABA, BA, Argentina), and 10% heat inactivated fetal bovine serum (FBS) (Thermo Fisher Scientific). Cells were seeded at 2 × 10^5^ cells/well in 24-well plates and maintained at 37 °C in a water-saturated 5% CO2 atmosphere. After that, cells were infected with a fresh bacterial inoculum in a multiplicity of infection (MOI) of 10. Two hours after the invasion, cells were washed with medium, prior to addition of cloxacillin, chitosan or different combination of them, to the culture for 24 h. Extracellular and intracellular bacterial viability were evaluated by plate count. Briefly, extracellular bacterial viability was determined by seeding the culture supernatant homogenized in TSA plates. After that, MAC-T cells were washed twice with PBS solution and incubated for 2 h with 100 µg/mL of gentamicin to eliminate extracellular bacteria. Intracellular bacterial viability was assayed after MAC-T cells lysed with 0.1% of Triton x100 and release the intracellular bacteria. Plates were incubated for 24 h at 37 °C and the viable bacteria were counted.

### Cytokine quantification

Cytokine secretion in culture supernatants of infected MAC-T cells in the presence of different conditions was assessed. IL-6 and IL-1β concentrations in culture supernatants were measured by specific anti-bovine ELISA kit (Thermo Fisher Scientific) following the manufacturer’s instructions.

### Mammalian cytotoxicity assay

MAC-T cells were cultured in the presence of medium or in the presence of cloxacillin, chitosan or their combinations, the cytotoxicity effects over them were measured using MTT assay. In all cytotoxicity assays, 10% of Triton X-100 has been used as positive control.

### Experimental design and statistical analysis

In order to control the strain variability, a two factorial design was developed. A factor was treatment and a second factor was strain. In each “treatment-strain” combination, the number of independent replicates used has been specified. Statistical analysis was performed using one and two-way ANOVA with Bonferroni post hoc test analysis. Mean ± SEM are represented in the graphs. Statistical tests and graphs were performed using the R soft (R Core Team, 2016) and GraphPad Prism 5.0 (GraphPad Software, Inc., CA, USA). P values *< 0.05, **< 0.01 and ***< 0.001 were considered significant in all analyses.

## Electronic supplementary material

Supplementary figures 1 and 2
