# Supplementary material for: Chitosan and cloxacillin combination improve antibiotic efficacy against different lifestyle of coagulase-negative Staphylococcus isolates from chronic bovine mastitis
Source: Sci Rep. 2018 Mar 23;8:5081. doi: 10.1038/s41598-018-23521-0 (PMC5865155; doi:10.1038/s41598-018-23521-0)
Supplement: Supplementary file 1 — Supplementary figures 1 and 2 [file 41598_2018_23521_MOESM1_ESM.pdf]

# Chitosan and cloxacillin combination improve antibiotic efficacy against different lifestyle of coagulase-negative *Staphylococcus* isolates from chronic bovine mastitis

María L. Breser; Verónica Felipe; Luciana P. Bohl; María S. Orellano; Paula Isaac; Agustín Conesa; Virginia E. Rivero; Silvia G. Correa; Ismael D. Bianco; Carina Porporatto.

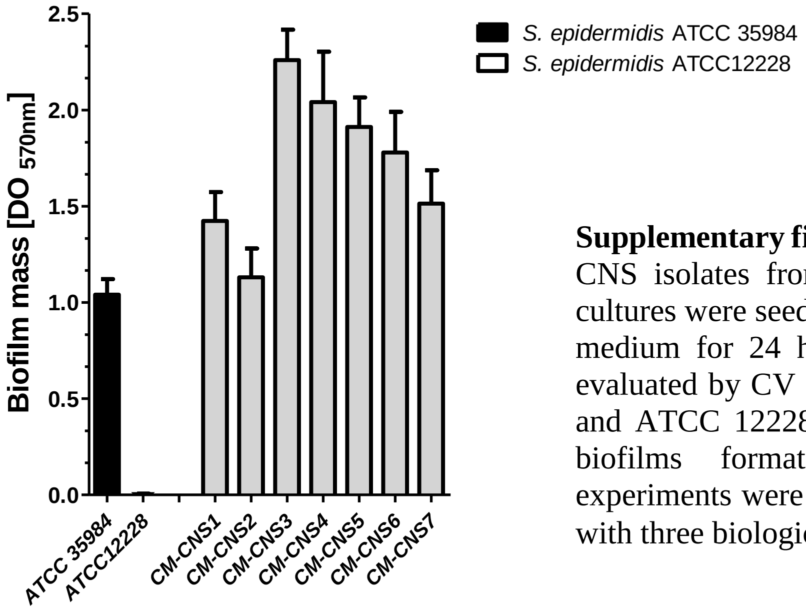


**Supplementary figure 1.** Bacterial biomass produced by CNS isolates from chronic bovine mastitis. Bacterial cultures were seeded in 96-well plates in the presence of medium for 24 hours at 37°C. Biofilm biomass was evaluated by CV staining. *S. epidermidis* ATCC 35984 and ATCC 12228 were used as positive and negative biofilms formation control, respectively. These experiments were performed in three independent times with three biological replicates.

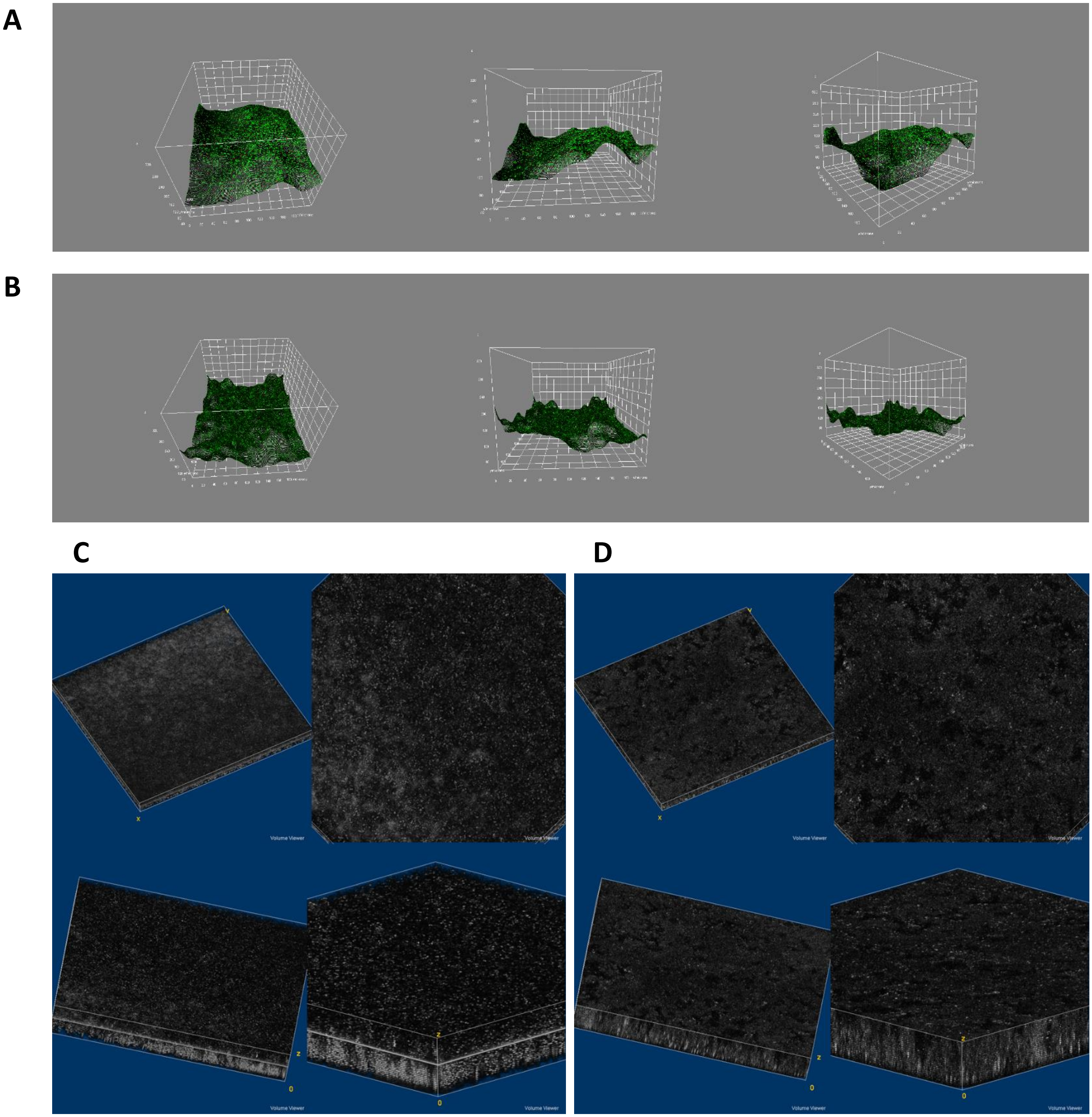


**Supplementary figure 2.** Bacterial biofilm 3D structure were evaluated by staining with SYTO9 and PI dyes and analysed by CLSM microscopy. CLSM images belong to CM-CNS7 isolation which was taken as representative. The other isolations showed similar efficiency results. A. Representative topography of Control condition. B. Representative topography of Chitosan condition. C. Different layers of the biomass volume of the bacterial biofilms in control condition. D. Different layers of the biomass volume of the bacterial biofilms under chitosan condition. Data were analysed using FIJI-ImageJ software.
